# Supplementary material for: Effects of mutating α-tubulin lysine 40 on sensory dendrite development
Source: J Cell Sci. 2017 Dec 15;130(24):4120–31. doi: 10.1242/jcs.210203 (PMC5769580; doi:10.1242/jcs.210203)
Supplement: Supplementary information [file joces-130-210203-s1.pdf]

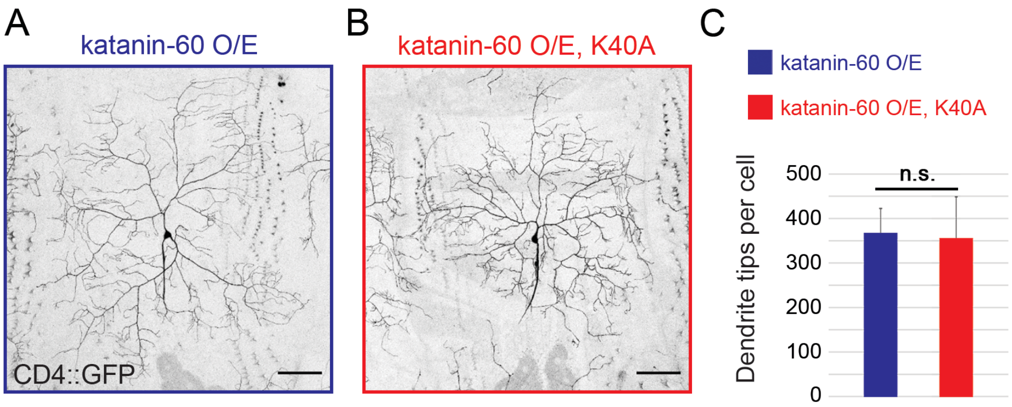

Supplemental Fig. 1. Dendrite branching is similar between neurons over-expressing katanin-60 in wild-type and αTub84B K40A animals. (A-C) Dendrite tip number is not significantly different between neurons over-expressing katanin-60 in a wild-type versus $\alpha Tub84B^{K40A}$ background. Scale bar: 100 µm. Experiments included age-matched controls that were imaged and analyzed in parallel. Dendrite tip analysis: 9 neurons over-expressing katanin-60 in a wild-type background and 7 neurons over-expressing katanin-60 in a $\alpha Tub84B^{K40A}$ mutant background were analyzed (mean ± SD). Statistical significance was evaluated using a two-tailed Student's t-test. n.s. = not significant.

Journal of Cell Science • Supplementary information
